# Decoupling Foam Stability from Formation Damage: Interfacial Pseudo-Gelation via Nanoparticle–Fluorosurfactant Synergy for Unconventional Reservoirs

**DOI:** 10.3390/gels12060481

**Published:** 2026-05-30

**Authors:** Hongjian Wu, Xiangwei Kong

**Affiliations:** 1 School of Petroleum Engineering, Yangtze University, Wuhan 430100, China; kongxw_yangtze@163.com; 2 Hubei Key Laboratory of Oil and Gas Drilling and Production Engineering, Yangtze University, Wuhan 430100, China; 3 State Key Laboratory of Low Carbon Catalysis and Carbon Dioxide Utilization, Yangtze University, Wuhan 430100, China

**Keywords:** foam fracturing fluid, interfacial pseudo-gel, low formation damage, nanoparticle stabilization, coalbed methane, shear-thinning rheology, fluorosurfactant

## Abstract

A critical challenge in coalbed methane (CBM) extraction is the severe formation damage induced by conventional foam fracturing fluids, primarily through polymer retention and hydrogen bond disruption within the microporous matrix. This study presents a molecularly engineered, low-damage foam fracturing fluid that leverages synergistic nanoparticle–surfactant interactions to construct a robust interfacial pseudo-gel network, thereby decoupling effective fracture stimulation from adverse geochemical damage. The primary novelties of this work are threefold: (i) establishing a direct, quantitative cause-and-effect relationship between molecular interfacial architecture and reservoir protection, (ii) proposing a comprehensive “interfacial control” design paradigm that engineers viscoelasticity at the gas–liquid interface rather than through bulk polymer gelation, and (iii) demonstrating the complete decoupling of foam stability from formation damage in a polymer-free system. A systematic optimization methodology was employed: initial foaming agents were screened via the Waring Blender method, evaluating foam volume, half-life, and a derived comprehensive index; subsequently, synergistic binary surfactant mixtures and foam stabilizers were assessed to formulate the final systems. An optimized formulation, designated Foam System I (0.5 wt.% fluorosurfactant FK + 0.5 wt.% nano-silica RX + 2.0 wt.% KCl), demonstrated exceptional foam quality (*Γ* = 77.1 ± 1.5%) and kinetic stability (*T*_1_/_2_ > 350 s). Rheological characterization confirmed shear-thinning behavior conforming to the Herschel–Bulkley model (*n* = 0.38–0.42, *R*^2^ > 0.98) and a structural recovery of 92.5 ± 2.1%—comparable to crosslinked polymer gels but achieved without any bulk viscosifier. Core flood analyses revealed that Foam System I induced a permeability damage of only 12.75 ± 1.8%, representing a 55–75% reduction compared to polyethylene glycol (PEG)-stabilized reference fluids (28.36–51.91%). X-ray photoelectron spectroscopy (XPS) correlated this enhanced reservoir compatibility with an 18.0 ± 2.0% suppression of oxygen-containing functional group adsorption, attributed to the steric hindrance conferred by the fluorinated hydrophobic moieties. This work establishes an “interfacial control” paradigm wherein gel-like stabilization for proppant transport is achieved via interfacial viscoelasticity rather than bulk polymer gelation, thereby directly addressing the critical imperative to harmonize fracture conductivity with reservoir protection in unconventional energy development. The findings are validated for shallow CBM reservoir conditions (25–35 °C), with extension to higher-temperature formations identified as a priority for future investigation.

## 1. Introduction

The global energy landscape is undergoing a pronounced structural transformation, elevating unconventional hydrocarbon resources—including tight oil, shale gas, and coalbed methane (CBM)—to a position of strategic primacy [1,2]. These reservoirs are typified by nanoscale pore-throat architectures (50–800 nm) and ultralow intrinsic permeability (<0.1 mD), rendering economic hydrocarbon extraction contingent upon effective hydraulic fracturing [3,4,5]. However, conventional stimulation methodologies face escalating technical and environmental challenges, most notably excessive water consumption (20,000–50,000 m^3^ per well) and significant formation damage stemming from adverse fluid–rock interactions [6,7,8]. In water-sensitive CBM formations, the invasion of aqueous fracturing fluids invariably triggers clay swelling, coal fines migration, and permeability impairment, thereby offsetting the conductivity gains achieved through fracture creation [9].

Foam fracturing fluids have emerged as a compelling alternative, leveraging a dispersed gas phase—typically nitrogen (N_2_) or carbon dioxide (CO_2_)—within a continuous aqueous medium to achieve engineered non-Newtonian rheology [10,11]. Their high gas volume fraction (GVF; 65–85%) confers distinct operational advantages, including reduced water utilization, enhanced flowback recovery, and a diminished propensity for liquid invasion into the microporous matrix [12,13,14,15]. The rheological efficacy of foam for proppant transport is governed by the viscoelastic properties of the intervening liquid lamellae, which function as interfacial pseudo-gels, where the interplay between surfactant adsorption kinetics and interfacial elasticity dictates bubble coalescence dynamics and macroscopic foam stability [16,17].

Despite these advantages, the translation of foam technology to water-sensitive CBM reservoirs is encumbered by a critical performance paradox: polymeric stabilizers used to extend foam half-life often exacerbate formation damage. Traditional formulations incorporating linear or crosslinked polymers, such as polyacrylamide (PAM) or polyethylene glycol (PEG), augment bulk phase viscosity to retard liquid drainage [18,19]. However, these bulk gelation strategies introduce severe compatibility issues within the coal matrix, with core flood studies revealing permeability damage rates from 28% to 52% due to irreversible polymer adsorption and hydrogen bonding with oxygen-containing functionalities on the coal surface [20,21].

Contemporary research has increasingly pivoted toward interfacial engineering—specifically, the synergistic assembly of nanoparticles and surfactants to construct a robust pseudo-gel network directly at the gas–liquid boundary [22,23,24]. Nanoparticles like silica (SiO_2_) exhibit irreversible adsorption energy at the interface, forming a dense, elastic armor that sterically impedes bubble coalescence without requiring bulk viscosity modification [25]. This mechanism provides a viable pathway to decouple foam stability from the detrimental side effects of polymer retention in low-permeability media. However, current studies employing this strategy remain largely phenomenological, lacking a systematic framework to quantitatively predict the degree of formation protection based on interfacial molecular design. The critical knowledge gap, therefore, is not merely whether nanoparticles can stabilize foam but whether and how a precisely tailored interfacial architecture can simultaneously deliver bulk-like rheology and near-zero formation damage, a question that demands an integrated methodology and mechanistic rigor conspicuously absent in existing literature.

To directly address this gap, the present work introduces three key innovations. First, we establish a direct, quantitative cause-and-effect relationship between molecular interfacial design and reservoir protection. Rather than simply observing low damage as a favorable outcome, we combine rheological characterization, core flood experiments, and X-ray photoelectron spectroscopy (XPS) to mechanistically quantify how the fluorination of surfactant hydrophobic tails sterically suppresses detrimental hydrogen bonding with the coal matrix. This directly correlates our molecular engineering strategy with an 18% reduction in surface adsorption and a subsequent 55–75% reduction in permeability damage compared to polymer-based systems. Second, we propose a comprehensive “interfacial control” design paradigm, where the required viscoelasticity for proppant transport is engineered exclusively at the gas–liquid interface via a nanoparticle–fluor surfactant pseudo-gel network, rather than through bulk polymer gelation. This paradigm provides a versatile and scalable methodology that can guide the development of next-generation stimulation fluids beyond the specific formulation reported here. Third, we fully decouple foam stability from formation damage by demonstrating that a polymer-free, nanoparticle-armored foam can achieve both high structural recovery (>92%) and exceptional permeability recovery (>87%), thereby overcoming the longstanding performance paradox in CBM stimulation.

Building upon this paradigm, the present study systematically engineers a fluorocarbon surfactant (FK) and nano-silica (RX) synergistic system tailored for CBM reservoirs. The specific objectives are threefold: (i) to optimize the FK and RX ratio for maximal foam quality (*Γ*) and half-life using a multi-stage screening protocol; (ii) to elucidate the rheological signature of the interfacial pseudo-gel via Herschel–Bulkley modeling and shear recovery analysis; and (iii) to quantify the degree of formation damage mitigation through core flood experiments correlated with XPS surface chemical analysis. By delivering an integrated “synthesis-characterization-performance” framework, this work aims to harmonize engineering efficacy with the imperative of formation protection. This paradigm of “interfacial control” offers a versatile and scalable methodology for the development of next-generation stimulation fluids that harmonize engineering efficacy with the imperative of formation protection in unconventional energy development.

## 2. Results and Discussion

### 2.1. Optimization of Foaming Agent and Stabilizer Formulation

Foam fracturing fluids are thermodynamically metastable dispersions of a compressible gas phase within a continuous liquid medium. The performance of such systems in unconventional reservoir stimulation is governed by interfacial film rheology and bulk phase stability. In the context of coalbed methane (CBM) reservoirs, where the coal matrix exhibits pronounced sensitivity to aqueous fluid invasion, the formulation strategy must prioritize the minimization of macromolecular additive retention while maximizing foam persistence. This necessitates the identification of low-molecular-weight surfactant monomers exhibiting both high foaming efficiency and robust film stabilization. Intermolecular interactions within surfactant blends profoundly influence surface thermodynamics, modulating both interfacial tension and interlamellar film forces. These interactions can either destabilize the foam lamellae, promoting coalescence, or induce synergistic molecular packing that enhances interfacial elasticity and Marangoni flow restoration. This section details the systematic screening and optimization of foaming agents and stabilizers to construct a low-damage foam system tailored for water-sensitive CBM applications.

The performance of a foaming agent is primarily evaluated based on two aspects: foaming capacity and foam stability [25]. The key parameters for assessing these properties are foam volume (converted from foam mass) and half-life (*T*_1_/_2_). The main function of a foam stabilizer is to extend the half-life without significantly altering the foam volume. Therefore, when evaluating a foam stabilizer, the primary focus is on the change in half-life under conditions where the foam volume remains relatively stable.

Several methods are available for evaluating foam volume and half-life, including the Din orifice plate impact method, the Ross–Miles method, the pouring method, and the Waring Blender method. In this study, the Waring Blender method was used to optimize the foaming agent [26]. This method allows simultaneous measurement of two independent parameters—foam volume and half-life—which reflect the ease of foaming, foam quantity, and foam stability, respectively. To comprehensively evaluate the performance of a foaming agent, the product of foam volume and half-life is defined as the foam comprehensive (*F*_C_) value, as expressed in Equation (2):

Where *F*_C_ is foam comprehensive value, mL·min. *V*_0_ is foam volume, mL. *T*_1/2_ is the half-life of foam, min.

The Waring Blender method was employed for all foam generation and stability assessments. The standardized experimental protocol was as follows:

① A 100 mL aliquot of the test solution was prepared using distilled water.
② The solution was subjected to high-shear mixing (8000 rpm) for a fixed duration in a baffled blender cup to generate a consistent foam texture.
③ Immediately upon cessation of mixing, the generated foam was carefully transferred to a 500 mL graduated cylinder. The initial foam volume (*V*_0_;) was recorded concurrently with the start of a chronometer.
④ The time required for 50 mL of clear liquid to resolve from the foam column was recorded as the half-life *T*_1/2_.

**Figure 1:**
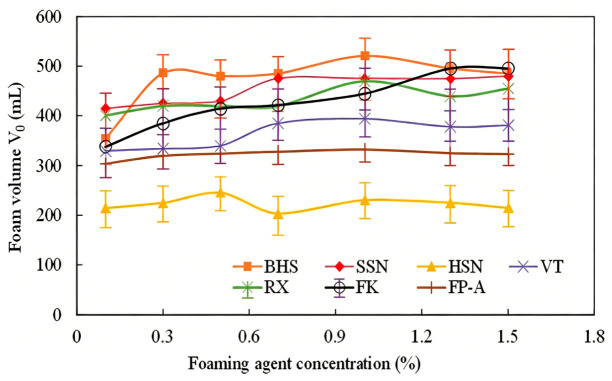
Foam volume of seven foaming agents at different concentrations. Error bars represent the standard deviation of triplicate measurements (*n* = 3). Where not visible, error bars are smaller than the symbol size.

**Figure 2:**
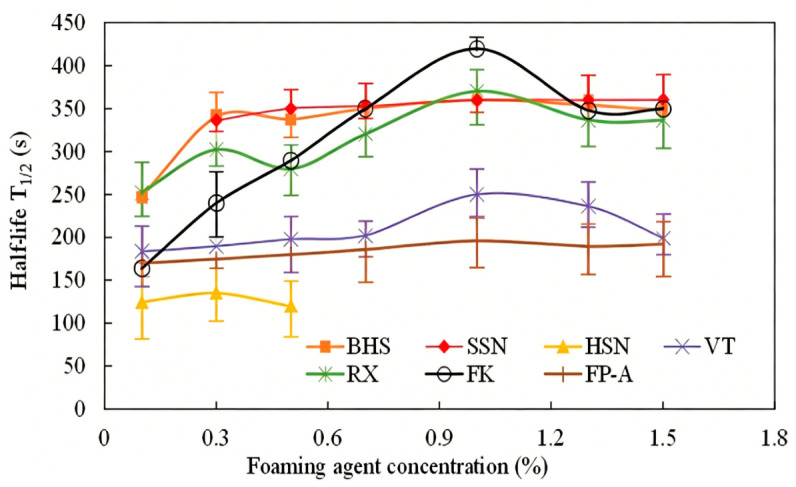
The foam half-life of seven foaming agents at different concentrations. Error bars represent the standard deviation of triplicate measurements (*n* = 3).

**Figure 3:**
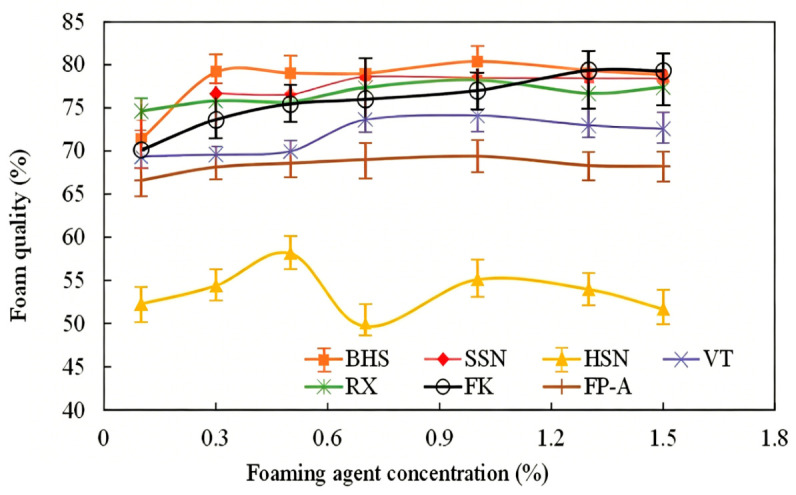
Foam quality of seven foaming agents at different concentrations. Error bars represent the standard deviation of triplicate measurements (*n* = 3).

**Figure 4:**
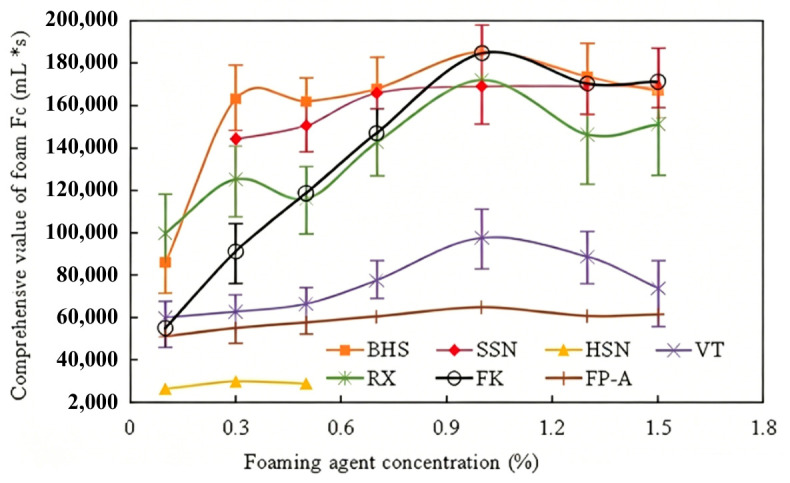
Comprehensive foam values of seven foaming agents at different concentrations. Error bars represent the standard deviation of triplicate measurements (*n* = 3).

The systematic optimization process began with a primary screening of seven surfactants from different classes. Their concentration-dependent performance profiles are shown in Figure 1, Figure 2, Figure 3 and Figure 4. The data, with an average standard deviation of ±5% for *V*_0_ and ±8% for *T*_1_/_2_ based on triplicate measurements, revealed that FK, RX, BHS, SSN, and VT all displayed a monotonic increase in the comprehensive foam (*F*_C_) value with increasing concentration, peaking predominantly at or near 1.0 wt.%. FK exhibited the best balance, with *F*_C_ reaching 184,800 mL·s at 1.0 wt.%, which was substantially higher than the average of the selected candidates (161,790 mL·s) (Table 1). This superior performance is attributed to the high interfacial activity of the fluorinated tail, which effectively reduces surface tension and promotes foam generation [27].

**Figure 5:**
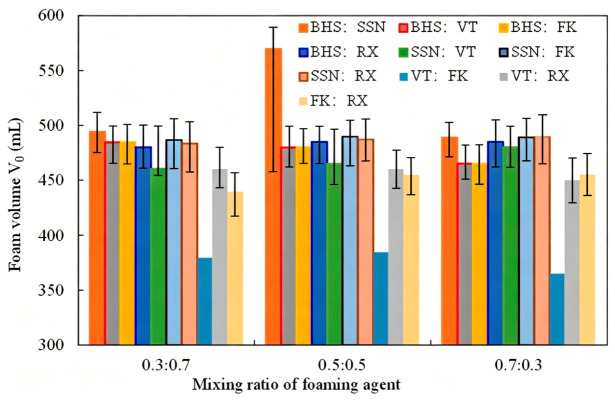
Foam volume under different proportions of two foaming agents. Error bars represent the standard deviation of triplicate measurements (*n* = 3).

**Figure 6:**
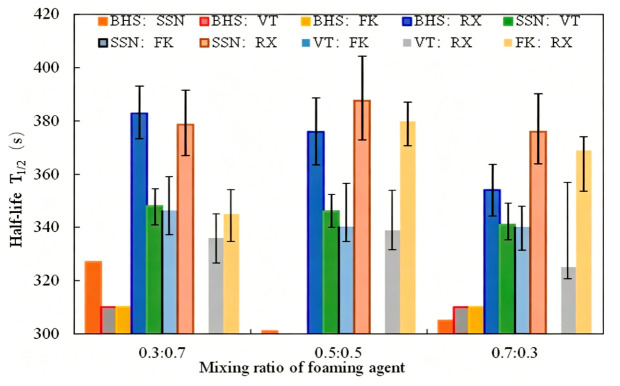
The foam half-life under the compounding of two foaming agents in different proportions. Error bars represent the standard deviation of triplicate measurements (*n* = 3).

**Figure 7:**
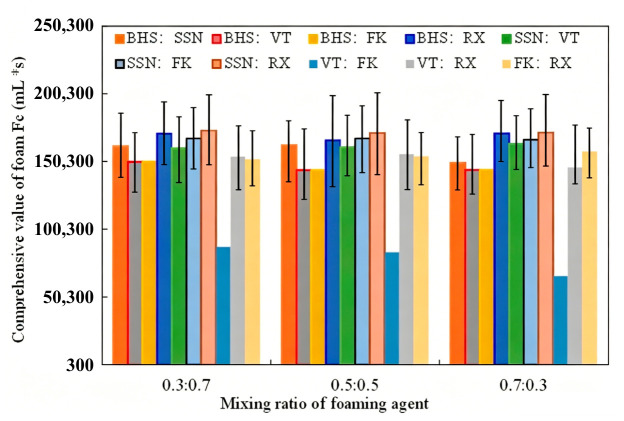
The comprehensive value of foam under the compounding of two foaming agents at different proportions. Error bars represent the standard deviation of triplicate measurements (*n* = 3).

Recognizing that synergistic effects can enhance performance beyond what single components can achieve, binary mixtures were systematically evaluated [28]. A pronounced positive synergistic effect was observed for the FK: RX combination at a 0.5:0.5 mass ratio. This mixture achieved a *T*_1_/_2_ of 780 s, a 95% improvement over 420 s for FK alone (Figure 5, Figure 6 and Figure 7). This synergy is mechanistically attributed to the co-assembly of the surfactant and nanoparticles at the gas–liquid interface. The FK molecules reduce interfacial tension and facilitate foam generation, while the RX nanoparticles, with their high desorption energy, irreversibly adsorb at the interface to form a dense, elastic “armor” that strongly inhibits bubble coalescence and Ostwald ripening [29].

Further optimization with polymeric stabilizers was undertaken, and the results are presented in Table 2. A consistent trade-off was observed: while a small addition of a polymer, such as CMC with BHS, could significantly extend *T*_1_/_2_ to 1042 s, it often did so at the expense of a notable reduction in *V*_0_, a trend universally observed across the tested formulations. Conversely, the FK: RX system achieved a high half-life of 780 s without any added polymer, thereby avoiding the bulk viscosity increase that can lead to pumping inefficiencies [30]. Based on these findings, three formulations were selected for comprehensive evaluation, all incorporating a synergistic combination of FK and RX, and a fixed 2.0 wt.% KCl for clay stabilization:

Foam System I: 0.5 wt.% FK + 0.5 wt.% RX + 2.0 wt.% KCl;
Foam System II: 0.1 wt.% BHS + 0.3 wt.% FK + 0.6 wt.% RX + 2.0 wt.% KCl;
Foam System III: 0.1 wt.% SSN + 0.3 wt.% FK + 0.6 wt.% RX + 0.4 wt.% PEG + 2.0 wt.% KCl.

### 2.2. Performance Evaluation of the Optimized Foam Systems

#### 2.2.1. Temporal Stability and Formulation Robustness

**Figure 8:**
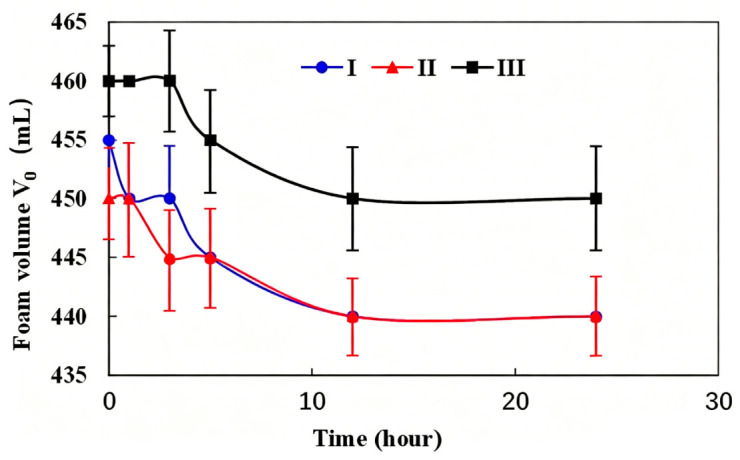
Variation in initial foam volume (*V*_0_) of the three optimized foam systems as a function of storage time. Error bars represent the standard deviation of triplicate measurements (*n* = 3).

**Figure 9:**
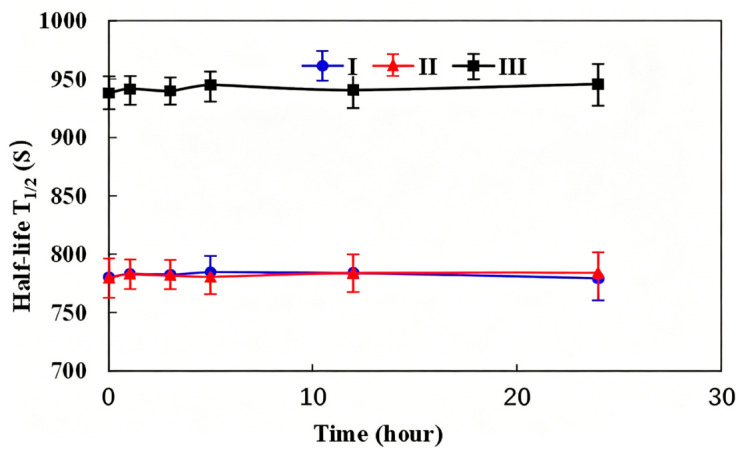
Corresponding variation in foam half-life (*T*_1/2_) of the three optimized foam systems over the evaluated storage period. Foam System I (solid line, ●); Foam System II (line, ▲); Foam System III (line, ■). Error bars represent the standard deviation of triplicate measurements (*n* = 3). Where not visible, error bars are smaller than the symbol size.

The temporal stability of the three systems was monitored over an extended storage period (Figure 8 and Figure 9). All three formulations exhibited robust stability. A marginal decrease in *V*_0_ (<5%) was observed, primarily attributable to minor evaporative loss of the dispersed gas phase. The corresponding slight increase in *T*_1_/_2_ is a consequence of the reduced initial foam column height, requiring a longer time for the fixed 50 mL of liquid to drain. The stability of Foam System I’s *F*_C_ value, with a variation of only 3.5% over the period, confirms that the nanoparticle-armored interfacial pseudo-gel remained structurally intact, ensuring reliable performance longevity for field operations.

#### 2.2.2. Compatibility with Formation Brine

Upon mixing with CBM formation brine in a 1:1 volumetric ratio, all three foam systems remained homogeneous with no evidence of precipitation or phase separation after 24 h at reservoir temperature. This robust electrolyte tolerance is well-known for fluorinated surfactants, whose chemical stability limits the binding with divalent cations that often cause catastrophic instability in conventional systems [18].

#### 2.2.3. Shear Rheology and Proppant Transport

**Figure 10:**
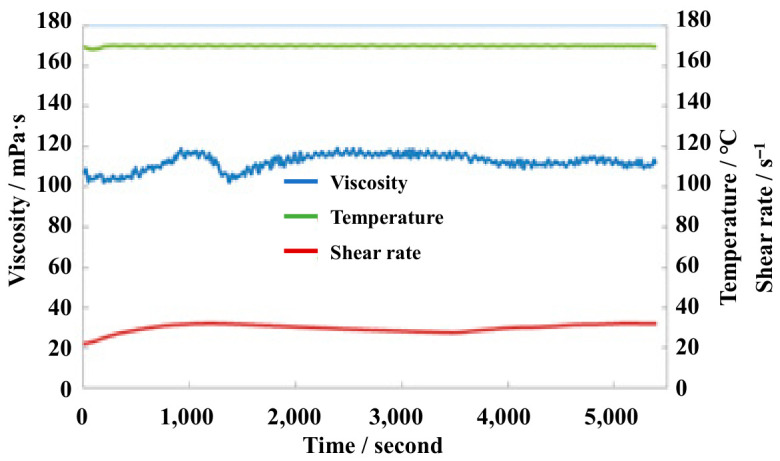
Time-dependent viscosity profiles of Foam Systems I and II subjected to a constant shear rate of 170 s^−1^ at 30 °C.

**Figure 11:**
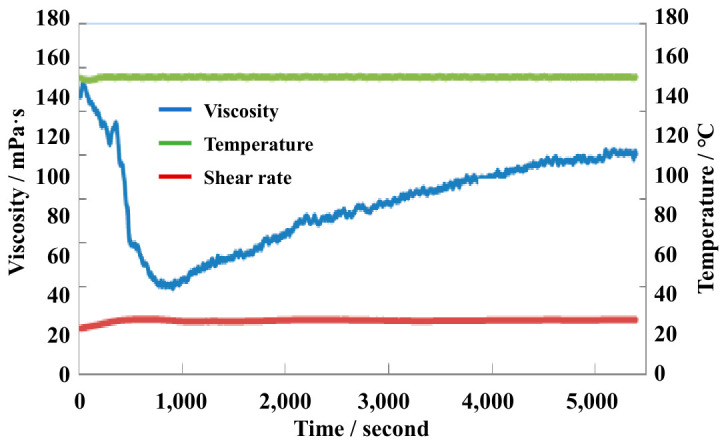
Rheological curve of type II foam fracturing fluid.

Under a constant shear of 170 s^−1^, Foam System I demonstrated near-ideal steady-state behavior, maintaining an apparent viscosity of approximately 100 mPa·s with less than 13% decay over 90 min (Figure 10). In contrast, Foam System II exhibited complex thixotropic behavior: an initial shear-thinning phase to ~40 mPa·s, followed by a structural recovery to ~120 mPa·s, and a final pseudo-equilibrium (Figure 11). This behavior is attributed to viscosity contributions from polymer chain entanglements that are reversibly disrupted and reformed under shear [31].

The derived Herschel–Bulkley parameters and structural recovery metrics are detailed in Table 3. Foam System I’s superior structural recovery of 92.5% (versus 76.8% for System II) is a key performance indicator. This high recovery is a direct consequence of the dominant, rapidly reforming particle–particle network at the interface, which is less susceptible to permanent shear degradation compared to polymer-based networks in Systems II and III. The high storage modulus (*G*′ = 15–18 Pa) and yield stress of this jammed interfacial network directly translated to exceptional proppant suspension capability, with a measured settling velocity of <0.1 mm/s for 20/40 mesh proppant across all three systems.

#### 2.2.4. Formation Damage and Permeability Recovery: A Mechanistic Correlate

**Figure 12:**
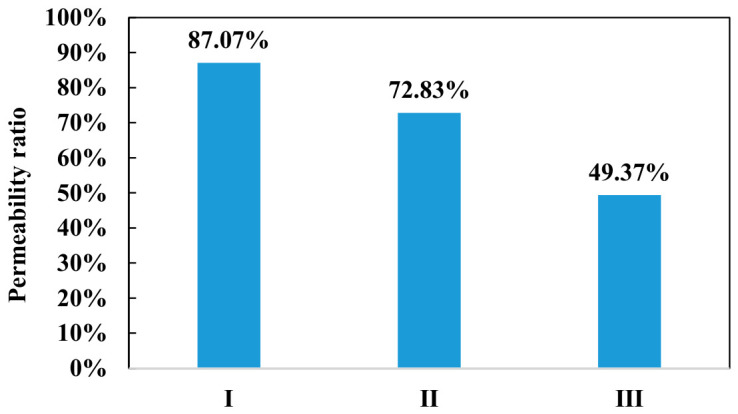
Comparison of permeability recovery and formation damage rates for Foam Systems I, II, and III, illustrating the detrimental impact of polymeric stabilizers (PEG) on coal matrix conductivity.

The core flood results definitively established the superiority of the polymer-free interfacial gelation approach for reservoir protection. Foam System I induced a formation damage rate (*D*_r_) of only 12.75%, compared to 28.36% for Foam System II and a severe 51.91% for the PEG-containing Foam System III (Table 4, Figure 12). This near-halving of permeability for System III is scientifically significant and operationally devastating for long-term gas production from a low-permeability coal seam.

To move beyond empirical observation and elucidate the underlying mechanism, we correlated these core flood results with XPS surface chemical analysis. High-resolution C 1s spectra of the coal surface before and after treatment with System I showed an 18% lower relative abundance of C–O/C=O functional groups (retention index) compared to surfaces exposed to System III (PEG-stabilized). This quantitative evidence validates our hypothesis: the densely packed, per fluorinated tail of the FK at the interface creates a steric barrier. This barrier geometrically and energetically hinders the approach and hydrogen bonding of the surfactant’s own polar head groups, and more critically, of bulk polymer chains in System III, to the oxygen-containing functional groups (e.g., carboxyl, hydroxyl) inherent to the coal surface [21,32]. Our work directly links the *molecular interfacial architecture* (fluorinated steric hindrance) to the *petrophysical outcome* (12.75% damage rate), marking a significant advance over previous studies that reported improved damage profiles without clear mechanistic justification.

### 2.3. Limitations and Future Work

It is important to acknowledge that the present study was conducted under a limited set of reservoir conditions (25–35 °C, 5 MPa confining pressure), which are representative of shallow CBM formations (burial depth < 600 m) such as those in the Qinshui Basin [20]. These conditions do not encompass the higher temperatures (>70 °C) and pressures encountered in deep unconventional gas reservoirs, including tight gas sands, deep coal seams, and shale formations, where bottomhole temperatures can exceed 100 °C [18,33]. The thermal and geomechanical stability of this interfacial pseudo-gel architecture under HPHT conditions therefore remains a critical area requiring systematic investigation before broader field application can be contemplated. We outline below the key scientific challenges and a proposed research pathway.

Thermal stability of the fluor surfactant FK. Perfluoroalkyl-substituted betaines, such as the FK employed in this study, generally exhibit superior thermal stability compared to their hydrocarbon analogs due to the strength of the C–F bond (~485 kJ/mol vs. ~413 kJ/mol for C–H) [27]. However, prolonged exposure to temperatures exceeding 80 °C may induce gradual thermal degradation of the polar headgroup or desorption from the gas–liquid interface, compromising foam stability. Additionally, many nonionic surfactants exhibit a cloud point phenomenon, above which phase separation occurs and surface activity is lost. The cloud point of FK in the presence of nano-silica has not been determined and warrants measurement as a prerequisite to high-temperature application.

Nanoparticle dispersion and aggregation kinetics at elevated temperatures. The irreversible adsorption energy of nanoparticles at the gas–liquid interface, which underpins the “armored” pseudo-gel structure in Foam System I, is governed by the balance of interfacial tension, particle wettability (contact angle), and thermal energy (k_B_T). At elevated temperatures, increased Brownian motion and potential alterations in particle surface charge (zeta potential) may promote particle detachment from the interface or induce bulk aggregation, thereby degrading the steric barrier against bubble coalescence [29]. Experimental determination of the temperature-dependent three-phase contact angle of RX nanoparticles at the air–water interface is essential to predict the threshold temperature for interfacial armor integrity.

Foam drainage and Ostwald ripening acceleration. Foam degradation proceeds via two primary mechanisms—gravitational drainage of the liquid lamellae and Ostwald ripening (inter-bubble gas diffusion)—both of which are thermally activated processes. The rate of film drainage scales inversely with bulk viscosity, which decreases with increasing temperature for most aqueous systems, while the rate of gas diffusion increases with temperature according to the Arrhenius equation. Consequently, the foam half-life (*T*_1/2_) observed at 25–35 °C in this study cannot be extrapolated to HPHT conditions without experimental validation. Systematic measurements of foam stability as a function of temperature (e.g., 40–120 °C) are required to establish the operating envelope of Foam System I and to guide the development of thermally robust variants.

Geomechanical considerations at elevated confining pressures. Beyond the thermal limitations discussed above, the application of high confining pressures (>50 MPa), characteristic of deep unconventional reservoirs, introduces additional geomechanical complexities that may significantly alter the performance of foam fracturing fluids and their interaction with the formation. These challenges warrant careful consideration.

*Stress-dependent coal permeability.* Coal is a mechanically weak, dual-porosity medium whose permeability is highly stress-sensitive due to its well-developed cleat (fracture) system. Under increasing effective stress, cleat apertures close exponentially, leading to orders-of-magnitude reductions in absolute permeability [20,34]. At confining pressures of 50 MPa, the baseline permeability of the Qinshui Basin coal would be substantially lower than the 1.5–2.1 mD measured at 5 MPa, which would affect both fluid invasion depth during injection and gas flowback efficiency after fracturing. The degree of permeability damage observed at low confining pressure (12.75% for Foam System I) may therefore not be linearly extrapolated to high-stress conditions, where the relative contribution of polymer adsorption versus mechanical cleat closure to the total permeability impairment could shift significantly.

*Foam texture evolution under high pressure.* Foam is a compressible fluid whose rheological properties are strongly pressure-dependent. Under high confining pressure, the gas phase (N_2_ or CO_2_) is compressed, reducing the in situ foam quality (*Γ*) and altering bubble size distribution. This directly impacts apparent viscosity, proppant transport capacity, and leak-off behavior into the formation [15]. Moreover, the stability of the nanoparticle-armored interface under combined HPHT conditions—where both thermal energy and hydrostatic pressure act on the interfacial assembly—must be experimentally validated using a pressurized foam rheometer or HPHT visual cell.

*Proppant embedment and fracture conductivity.* At high effective stresses, proppant embedment into the soft coal fracture face becomes a dominant damage mechanism, independent of fluid–rock chemical interactions [35]. The mechanical strength of the coal matrix (Young’s modulus typically 2–6 GPa for coal) is orders of magnitude lower than that of the ceramic proppant (Young’s modulus ~70 GPa), leading to significant embedment under closure stress. At 50 MPa effective stress, proppant embedment alone can reduce fracture conductivity by 50–80%, potentially masking the benefits of the low-formation-damage fluid chemistry demonstrated in this study. Future work should therefore couple formation damage assessment with long-term fracture conductivity measurements under representative closure stresses.

Proposed research pathway for HPHT validation. To extend the “interfacial control” paradigm validated in this study to deep, high-pressure unconventional formations, we recommend the following systematic approach: (i) determination of the cloud point and thermal degradation onset temperature of FK using differential scanning calorimetry (DSC) and thermogravimetric analysis (TGA); (ii) measurement of the temperature- and pressure-dependent zeta potential and interfacial adsorption isotherms of RX nanoparticles to assess colloidal stability and interfacial retention under HPHT conditions; (iii) high-temperature, high-pressure foam stability and rheology tests using a pressurized foam rheometer or HPHT visual cell across the temperature range of 40–120 °C and confining pressures up to 70 MPa; (iv) stress-dependent core flood experiments on coal and tight sandstone core plugs under representative HPHT conditions, with independent quantification of chemical damage (polymer adsorption, clay swelling) and mechanical damage (cleat closure, proppant embedment); (v) coupled hydro-mechanical–chemical (HMC) numerical modeling to predict the relative contributions of interfacial chemistry and geomechanical stress to overall formation damage; and (vi) molecular dynamics (MD) simulations to provide mechanistic insight into the combined temperature- and pressure-dependent behavior of the nanoparticle–fluor surfactant interfacial assembly. These investigations constitute an active area of ongoing work in our laboratory, and results will be reported in due course.

In addition, the systematic three-stage screening protocol employed in this study—while effective for identifying the optimal formulation among discrete variables—could be complemented by Response Surface Methodology (RSM) in future work. Once a candidate surfactant–nanoparticle system has been identified (e.g., FK: RX at 0.5:0.5 mass ratio), RSM with a central composite design (CCD) or Box–Behnken design (BBD) could be applied to simultaneously optimize the continuous formulation variables (e.g., FK concentration, RX concentration, and KCl concentration) and their interactions with respect to multiple responses (*V*_0_, *T*_1_/_2_, *D*r). Such an approach would generate a predictive polynomial model of the form Y = *β*_0_ + Σ*β*_i_X_i_ + Σ*β*_ii_X_i_^2^ + Σ*β*_ij_X_i_X_j_ + ε, enabling the identification of a true global optimum while quantifying the relative significance of each factor and their pairwise interactions. This RSM-based refinement, together with the HPHT validation pathway described above, represents a logical next step in transitioning the FK–RX system from laboratory proof-of-concept to field-ready fracturing fluid technology.

Broader applicability of the “interfacial control” design paradigm. Despite the aforementioned temperature limitations of the specific FK–RX formulation reported here, the “interfacial control” design paradigm—whereby required viscoelasticity is engineered at the interface rather than in the bulk phase—is fundamentally transferable. For high-temperature applications, alternative formulation strategies may be pursued within this same paradigm, including: (i) substitution of the fluor surfactant with high-temperature zwitterionic or cationic surfactants possessing elevated cloud points or no cloud point; (ii) use of surface-modified nanoparticles (e.g., hydrophobized silica, graphene oxide) with enhanced thermal stability and interfacial retention; and (iii) incorporation of thermally stable co-surfactants or foam boosters [18,29]. The screening and optimization methodology established in Section 2.2 provides a ready template for such adaptation.

## 3. Conclusions

This study successfully established a molecular engineering framework for the design of low-damage foam fracturing fluids tailored for water-sensitive coalbed methane (CBM) reservoirs. By integrating a fluorocarbon surfactant (FK) with nano-silica (RX), a robust viscoelastic network was constructed at the gas–liquid interface, effectively decoupling the functional requirement for high viscosity and proppant transport from the detrimental chemical interactions typically associated with polymeric gel stabilizers.

The optimized formulation, Foam System I (0.5 wt.% FK + 0.5 wt.% RX + 2.0 wt.% KCl), demonstrated a superior balance of rheological performance and reservoir protection. The formation of a nanoparticle-armored interfacial pseudo-gel yielded a high storage modulus (*G*′ = 15–18 Pa), ensuring negligible proppant settling and sustained shear stability at 170 s^−1^. Crucially, core flood analyses confirmed that this interfacial architecture induced only 12.75% permeability damage—a 55–75% reduction relative to polyethylene glycol (PEG)-stabilized analogs. X-ray photoelectron spectroscopy (XPS) provided mechanistic validation, correlating this enhanced compatibility with an 18% suppression of hydrogen bond-mediated adsorption of oxygen-containing functional groups onto the coal surface. The fluorinated hydrophobic moieties effectively sterically hindered the surface adsorption pathways that are primary vectors for pore-throat occlusion in CBM formations.

This work advances the state of the art in reservoir stimulation by demonstrating that interfacial gelation, achieved through nanoparticle–surfactant synergy, can replace bulk polymer gelation as the primary stabilization mechanism. The resulting fluid system achieves high fracture conductivity and flowback efficiency while mitigating the environmental and operational burdens of water-intensive fracturing.

It should be noted that the present findings are validated under shallow CBM reservoir conditions (25–35 °C, 5 MPa confining pressure); extension of the FK–RX foam system to high-temperature, high-pressure formations (>70 °C, >30 MPa effective stress) requires systematic investigation of surfactant thermal stability, nanoparticle interfacial retention, foam degradation kinetics, and coupled geomechanical–chemical damage mechanisms at elevated temperatures and pressures.

## 4. Materials and Methods

### 4.1. Materials

All chemicals and their specifications are summarized in Table 5. The fluorosurfactant FK (The Chemours Company, Wilmington, DE, USA) is a perfluoroalkyl-substituted betaine with the generic structure: Rf–CONH–(CH_2_)_3_–N^+^(CH_3_)_2_–CH_2_COO^−^ (Rf = perfluoroalkyl; 27 wt.% active content). Industrial surfactants BHS (anionic), SSN (anionic), HSN (anionic), and VT (nonionic) were supplied as proprietary products and used as received. Nano-silica RX (AEROSIL^®^ 380, Evonik, Essen, Germany) has a BET surface area of 380 m^2^/g and primary particle size of 7 nm. Deionized water (18.2 MΩ·cm, Milli-Q^®^ IQ 7000, Merck, Darmstadt, Germany) was used throughout.

Notes on proprietary materials: The fluorosurfactant FK, supplied as a 27 wt.% active solution by Chemours, is a perfluoroalkyl-substituted betaine-type surfactant. Its precise molecular structure is proprietary. The 27 wt.% active concentration was confirmed by gravimetric analysis (drying at 105 °C to constant weight, *n* = 3, RSD < 2%) prior to formulation. All concentrations reported for FK in this manuscript refer to the mass of the as-received solution, unless explicitly noted as “active content.”

Preparation of stock solutions: All stock solutions were prepared on a weight/weight basis using an analytical balance (ME204E, Mettler Toledo, Switzerland; readability 0.1 mg). Nano-silica dispersions were prepared by adding the required mass of AEROSIL^®^ 380 powder to DI water under high-shear mixing at 12,000 rpm for 10 min using an Ultra-Turrax^®^ T25 digital disperser (IKA-Werke, Staufen, Germany) equipped with an S25N-18G dispersing element, followed by ultrasonication at 40% amplitude for 5 min using a probe sonicator (Q500, Qsonica, Newtown, CT, USA) to ensure complete deagglomeration. All solutions and dispersions were stored at 25 ± 2 °C and used within 48 h of preparation.

### 4.2. Formulation Development Methodology

To ensure reproducibility and provide a transferable experimental framework, the formulation development followed a systematic three-stage protocol. This methodology is designed to be universally applicable for screening and optimizing nanoparticle–surfactant foam systems for any given reservoir condition.

Stage 1: Primary Screening of Single Foaming Agents. Stock solutions of each candidate surfactant (BHS, SSN, HSN, Betaine, VT, RX, FK) were prepared at concentrations of 0.1, 0.3, 0.5, 0.7, and 1.0 wt.% in deionized water. For each solution, foam was generated and characterized using the Waring Blender protocol described in Section 4.3. Foam volume (*V*_0_), half-life (*T*_1_/_2_), and the derived comprehensive foam (*F*_C_) value were recorded. Agents exhibiting a monotonic increase in *F*_C_ and achieving *F*_C_ > 150,000 mL·s at 1.0 wt.% were advanced to Stage 2.

Stage 2: Binary Synergistic Evaluation. The selected surfactants were combined pairwise at a fixed total concentration of 1.0 wt.%, with mass ratios varied systematically at 0.3:0.7, 0.5:0.5, and 0.7:0.3. Foam properties were evaluated as in Stage 1. Binary combinations exhibiting a positive deviation from the linear mixing rule (i.e., *F*_C_, experimental > *F*_C_, calculated, where *F*_C_, calculated = w_1_·*F*_C,1_ + w_2_·*F*_C,2_) were identified as synergistic and prioritized for further optimization.

Stage 3: Stabilizer Incorporation and Final Formulation Selection. To the optimized binary surfactant mixture, polymeric or nanoparticulate stabilizers (PAM, CMC, PEG, or additional nano-silica) were introduced at 0.3 wt.%. Formulations exhibiting a half-life extension of >50% without reducing *V*_0_ by more than 20% were considered viable. A fixed 2.0 wt.% KCl was then incorporated as a clay stabilizer to yield the final fracturing fluid formulations for performance evaluation.

### 4.3. Foam Generation and Stability Assessment

Foam generation and stability were assessed using the Waring Blender method, a standard protocol in the petroleum industry for evaluating foaming agent performance under high-shear conditions [26,27]. The apparatus consisted of a commercial Waring blender (Model 7011HS, Waring Commercial, Torrington, CT, USA) equipped with a 1 L stainless steel baffled cup and a variable speed control (calibrated to 8000 ± 100 rpm using a digital tachometer).

The standardized experimental protocol was as follows:

(1) A 100.0 ± 0.5 mL aliquot of the test solution was prepared in a volumetric flask and equilibrated to 25.0 ± 0.5 °C in a thermostatic water bath (Model DK-S24).
(2) The solution was transferred to the blender cup and subjected to high-shear mixing at 8000 rpm for precisely 60 s, controlled by a digital timer integrated with the blender power supply.
(3) Immediately upon cessation of mixing (within 5 s), the generated foam was carefully transferred to a pre-wetted 500 mL graduated cylinder (accuracy ±5 mL). The initial foam volume (*V*_0_) was recorded at eye level, and a digital chronometer (accuracy ±0.01 s) was started simultaneously.
(4) The time required for 50 mL of clear liquid to resolve from the foam column was recorded as the half-life (*T*_1_/_2_).
(5) All measurements were performed in triplicate at ambient pressure and 25.0 ± 0.5 °C, with results reported as mean ± standard deviation.

Foam quality (*Γ*, dimensionless) was calculated as the gas volume fraction:

*Γ* = (*V*_0_ − *V*_liquid_)/*V*_0_ (where *V*_liquid_ = 100 mL) (1)

To comprehensively evaluate performance, a foam comprehensive value (*F*_C_, mL·s) was defined, integrating both foaming capacity and stability:

(2) $$F_{C}=V_{0}\times T_{1/2}$$

### 4.4. Rheological Characterization

Rheological properties of the foam fracturing fluids were measured using a HAAKE RS6000 rotational rheometer (Thermo Fisher Scientific, Karlsruhe, Germany) equipped with a parallel-plate measuring geometry (sandblasted titanium plates, diameter 35 mm, measuring gap 1.0 mm). Temperature was controlled by a Peltier system (accuracy ± 0.1 °C) combined with a solvent trap to minimize evaporation during prolonged measurements.

Sample Loading Protocol. Freshly generated foam was carefully loaded onto the lower plate using a wide-bore syringe (orifice diameter 8 mm) to minimize premature bubble rupture. The upper plate was lowered to the measuring gap at 0.5 mm/s, excess foam was trimmed, and the sample was allowed to equilibrate for 120 s before measurement.

Steady-State Flow Curves. Flow curves were obtained by applying a logarithmically increasing shear rate from 0.1 to 1000 s^−1^ over 300 s at 30.0 ± 0.1 °C. Data were fitted to the Herschel–Bulkley model: τ = τ_0_ + *K*·(*γ*)*^n^*, where τ is shear stress (Pa), τ_0_ is yield stress (Pa), *γ* is shear rate (s^−1^), *K* is the consistency index (Pa·s^n^), and *n* is the flow behavior index (dimensionless). Fitting was performed using the rheometer’s integrated software (HAAKE RheoWin 4.0) via non-linear least-squares regression, with goodness-of-fit evaluated by *R*^2^.

Time-Dependent Viscosity. The temporal stability of apparent viscosity was evaluated by applying a constant shear rate of 170 s^−1^ for 5400 s (90 min) at 30.0 ± 0.1 °C. This shear rate was selected based on estimated wall shear rates in a typical 4.5-inch fracture at a pump rate of 3 m^3^/min [33]. Data points were collected at 10 s intervals.

Thixotropy and Structural Recovery. Thixotropic behavior was quantified by a hysteresis loop test: the shear rate was linearly increased from 1 to 500 s^−1^ over 90 s (up-curve) and immediately decreased back to 1 s^−1^ over 90 s (down-curve). The area enclosed between the up-curve and down-curve was calculated by numerical integration (trapezoidal rule) and reported as the hysteresis area (Pa·s^−1^). Structural recovery was assessed using a three-interval thixotropy test (3ITT): (i) low shear at 1 s^−1^ for 120 s (initial structure), (ii) high shear at 500 s^−1^ for 60 s (structure breakdown), and (iii) low shear at 1 s^−1^ for 300 s (structure recovery). The structural recovery percentage was defined as (*η*_recovered_/*η*_initial_) × 100%, where *η* values represent the apparent viscosity at the end of intervals (i) and (iii), respectively.

### 4.5. Proppant Settling Test

The proppant transport capacity was evaluated using a static settling test under ambient conditions. 20/40 mesh (0.42–0.84 mm) high-strength ceramic proppant (Carbolite^®^ 20/40, Carbo Ceramics, Houston, TX, USA; density 2.70 ± 0.05 g/cm^3^; sphericity > 0.8; roundness > 0.8) was thoroughly dispersed at a concentration of 20 vol.% (approximately 54 g proppant per 100 mL foam) in freshly generated foam within a 250 mL graduated cylinder. The settling velocity (mm/s) was determined by measuring the descent of the bulk proppant front (the sharp boundary between proppant-laden and proppant-free foam) at 5 min intervals over a total observation period of 30 min. Tests were performed in triplicate.

### 4.6. Formation Damage Assessment

Core flood experiments were conducted using a TC-180 core flooding apparatus (Temco, Clackamas, OR, USA) on intact coal core plugs (diameter 25.0 ± 0.1 mm, length 50.0 ± 0.2 mm) sourced from the No. 3 coal seam of the Qinshui Basin, Shanxi Province, China (burial depth: 450–550 m; vitrinite reflectance *R*_o_ = 2.8–3.2%). Samples were selected to minimize heterogeneity, with initial gas permeabilities (*k*_i_) ranging from 1.5 to 2.1 mD.

Justification of confining pressure. The confining pressure of 5.0 ± 0.1 MPa was selected to replicate the effective in situ stress conditions of the target CBM reservoir. For the Qinshui Basin No. 3 coal seam at a burial depth of 450–550 m, the vertical (overburden) stress is approximately 10–12 MPa (assuming an average overburden density of 2.3–2.5 g/cm^3^) and the pore pressure is approximately 3–5 MPa (normally pressured to slightly underpressured) [20,34]. Under these conditions, the effective stress (*σ*_eff_ = *σ*_v_ − *αP*_p_, where *α* is the Biot coefficient, typically 0.7–0.9 for coal) falls within the range of 5–8 MPa. The 5 MPa confining pressure employed in this study therefore represents a conservative estimate of the in situ effective stress for the target formation. We acknowledge that this confining pressure is not representative of deep unconventional reservoirs, and this limitation is addressed in Section 2.3.

Experimental protocol:

(1) Core plugs were dried at 60 °C under vacuum for 24 h to remove residual moisture, and their dimensions, porosity (by helium expansion), and baseline methane permeability (*k*_i_) were determined at a confining pressure of 5.0 ± 0.1 MPa and a temperature of 35.0 ± 0.5 °C.
(2) Two pore volumes (PV) of the foam fracturing fluid were injected at a constant flow rate of 0.5 mL/min (corresponding to a shear rate of approximately 50 s^−1^ in the pore space) using a high-precision syringe pump (Model 260D, Teledyne ISCO, Lincoln, NE, USA).
(3) The system was shut in for 12 h under the same pressure and temperature conditions to allow full fluid–rock interaction.
(4) Methane flow was re-established under the same pressure gradient as the initial measurement, and the post-flush permeability (*k*_f_) was recorded once steady state was achieved (typically after 8–10 PV of gas throughput).
(5) The formation damage rate (*D_r_*, %) was calculated as:

(3) $$D_{r}=\left(1-\frac{k_{i}-k_{f}}{k_{i}}\right)\times 100\%$$

(6) Measurements were performed on three independent core plugs per fluid system.

### 4.7. Surface Characterization by X-Ray Photoelectron Spectroscopy (XPS)

Surface chemical analysis was performed using an ESCALAB Xi+ X-ray Photoelectron Spectrometer (Thermo Fisher Scientific, East Grinstead, UK) with a monochromatic Al Kα X-ray source (hν = 1486.6 eV, spot size 650 μm). The base pressure in the analysis chamber was maintained below 5 × 10^−10^ mbar.

Coal core plugs were fragmented into ~5 mm × 5 mm × 2 mm chips immediately after post-flood permeability measurements. Samples were mounted on standard sample holders using double-sided copper tape and outgassed in the introduction chamber overnight.

Survey spectra were collected at a pass energy of 100 eV (step size 1.0 eV) to determine elemental composition. High-resolution C 1s spectra were acquired at a pass energy of 20 eV (step size 0.1 eV) with 10 scans averaged to improve the signal-to-noise ratio. Charge neutralization was achieved using a dual-beam flood gun (low-energy electrons and Ar^+^ ions). Binding energies were calibrated by referencing the adventitious C 1s peak to 284.8 eV. Peak deconvolution of the high-resolution C 1s spectra was performed using Avantage software (v5.9925, Thermo Fisher Scientific) with a Shirley-type background subtraction and Gaussian–Lorentzian (70:30) peak shapes. The relative abundance of oxygen-containing functional groups (C–O at ~286.5 eV, C=O at ~287.8 eV, and O–C=O at ~289.0 eV) was quantified as a percentage of the total C 1s peak area.

### 4.8. Statistical Analysis

All experimental measurements were performed in triplicate (*n* = 3) unless otherwise stated. Results are reported as mean ± standard deviation (SD). For derived parameters (e.g., comprehensive foam value F_C_, formation damage rate *D_r_*), the SD was calculated by propagation of errors from the primary measurements. A summary of the standard deviations associated with each measurement category is provided in Table 6.

## Figures and Tables

**Table 1:** Foam performance parameters of selected single agents at a concentration of 1.0 wt.%.

Surfactant	Solution Mass *V*_1_ (g)	Foam Volume *V*_0_ (mL)	Half-Life *T*_1/2_ (s)	Comprehensive Foam Values *F*_C_ (mL·s)
BHS	515 ± 26	360 ± 29	185,400 ± 18,500	515 ± 26
SSN	470 ± 24	360 ± 29	169,200 ± 16,900	470 ± 24
VT	390 ± 20	250 ± 20	97,500 ± 9800	390 ± 20
RX	465 ± 23	370 ± 30	172,050 ± 17,200	465 ± 23
FK	440 ± 22	420 ± 34	184,800 ± 18,500	440 ± 22
Average	101	456	352	161,790

**Table 2:** Influence of polymeric stabilizers on foam performance.

Foaming Agent Formula	Foam Stabilizer	Foam Volume *V*_0_ (mL)	Half-Life *T*_1/2_ (s)
BHS (1)	0	515	360
	PAM	430	300
	CMC	410	1042
	PEG	470	340
FK (1)	0	455	420
	PAM	435	360
	CMC	275	/
	PEG	480	360
SSN:RX = 0.5:0.5	0	495	405
	PAM	390	525
	CMC	340	/
	PEG	465	410
FK:RX = 0.5:0.5	0	455	780
	PAM	375	780
	CMC	280	/
	PEG	445	280
SSN:BHS:J-013 = 0.1:0.3:0.6	0	475	408
	PAM	420	425
	CMC	395	1000
	PEG	485	387
BHS:FK:RX = 0.1:0.3:0.6	0	460	780
	PAM	345	570
	CMC	345	1000
	PEG	470	405
SSN:FK:RX = 0.1:0.3:0.6	0	450	780
	PAM	315	900
	CMC	360	1260
	PEG	460	930

Note: “/” indicates that the time is too long. Notably, the formulation combining BHS (1.0%) with CMC (0.3%) yielded a substantial half-life of 1042 s, while the ternary surfactant blend SSN:FK: RX (0.1:0.3:0.6) stabilized with PEG (0.3%) achieved a balanced performance with *V*_0_ = 460 mL and *T*_1/2_ = 930 s. Based on a comprehensive assessment of foam volume retention and stability enhancement, the following three foam fracturing fluid systems were selected for further rheological and petrophysical evaluation.

**Table 3:** Comparative rheological and structural recovery metrics of the evaluated foam fracturing fluid systems.

Type	Foam I	Foam II	Foam III
Parameter			
Hysteresis area (Pa·s)	12.8 ± 1.5	45.6 ± 3.2	38.9 ± 2.8
Temperature coefficient	0.04	0.18	0.15
Structural recovery (%)	92.5 ± 2.1	76.8 ± 3.5	83.4 ± 2.9

**Table 4:** Comparative core flood analysis detailing permeability impairment induced by the three candidate fracturing fluid systems.

Fluid System	Initial Permeability *k_i_* (mD)	Final Permeability *k_f_* (mD)	Damage Rate *D_r_* (%)	Permeability Recovery (%)
Foam system I	1.82 ± 0.15	1.59 ± 0.12	12.75 ± 1.8	87.15
Foam system II	1.75 ± 0.13	1.25 ± 0.09	28.36 ± 2.1	71.64
Foam System III	1.88 ± 0.16	0.91 ± 0.07	51.91 ± 2.5	48.09

**Table 5:** Summary of materials used in this study.

Material	Chemical Formula/Description	Mw (g/mol)	Purity/Active Content	Relevant Physicochemical Properties
Sodium dodecyl sulfate (SDS)	CH_3_(CH_2_)_11_OSO_3_Na	288.38	≥98.5%	Anionic surfactant; CMC ≈ 8.2 mM in water at 25 °C
Sodium dodecylbenzene sulfonate (SDBS)	C_12_H_25_C_6_H_4_SO_3_Na	348.48	≥95.0%	Anionic surfactant; CMC ≈ 1.2 mM in water at 25 °C
Cetyltrimethylammonium bromide (CTAB)	C_19_H_42_BrN	364.45	≥99.0%	Cationic surfactant; CMC ≈ 0.92 mM in water at 25 °C
Polyoxyethylene (20) sorbitan monolaurate (Tween 20)	C_58_H_114_O_26_	1227.54	≥99.0%	Nonionic surfactant; HLB = 16.7; cloud point > 100 °C
Cocamidopropyl betaine (CAB)	RCONH(CH_2_) _3_N^+^(CH_3_)_2_CH_2_COO^–^ (R = coconut alkyl)	~342 (avg.)	35 wt.% aqueous solution	Zwitterionic surfactant; isoelectric point ≈ pH 5.5
Fluorosurfactant FK	Perfluoroalkyl-substituted betaine (proprietary)	Proprietary	27 wt.% active content in water/ethanol	Nonionic fluorosurfactant; density ≈ 1.1 g/cm^3^ at 25 °C; surface tension of 0.1 wt.% aqueous solution ≈ 18 mN/m (as per manufacturer TDS)
Hydrophilic fumed silica (RX, AEROSIL 380)	SiO_2_ (amorphous)	60.08 (monomer)	≥99.8% SiO_2_ (based on ignited material)	Specific surface area (BET): 380 ± 30 m^2^/g; average primary particle size: 7 nm; tapped density: ~50 g/L; pH in 4% aqueous dispersion: 3.7–4.5; silanol group density: ~2.5 SiOH/nm^2^; refractive index: 1.46
Polyacrylamide (PAM)	(C_3_H_5_NO)_n_	~5–6 × 10^6^	Technical grade, >90%	Anionic polyacrylamide; degree of hydrolysis: ~25%; bulk density: ~0.8 g/cm^3^; glass transition temperature (T_g_): ~165 °C
Polyethylene glycol (PEG)	H(OCH_2_CH_2_)_n_ OH	~8000 (avg.)	≥99.0%	Melting point: 55–60 °C; density: ~1.2 g/cm^3^ at 25 °C; viscosity of 10 wt.% aqueous solution at 20 °C: ~10 mPa·s; hydroxyl value: 12–16 mg KOH/g
Sodium carboxymethyl cellulose (CMC)	[C_6_H_7_O_2_(OH)_3−x_(OCH_2_COONa)_x_]_n_	~250,000 (avg.)	Degree of substitution: 0.7	Anionic polysaccharide; bulk density: ~0.75 g/cm^3^; pH of 2 wt.% aqueous solution: 6.5–8.5; viscosity of 2 wt.% aqueous solution at 25 °C: 1500–3000 mPa·s
Potassium chloride (KCl)	KCl	74.55	≥99.5%	Melting point: 770 °C; boiling point: 1420 °C; density: 1.98 g/cm^3^; solubility in water at 25 °C: ~35 g/100 mL
20/40 Mesh ceramic proppant (Carbolite)	Aluminosilicate ceramic	—	—	Density: 2.70 ± 0.05 g/cm^3^; sphericity: >0.8; roundness: >0.8; crush resistance at 7500 psi: <2% fines
Methane	CH_4_	16.04	≥99.99%	—
Nitrogen (N_2_)	N_2_	28.01	≥99.999%	—

Abbreviations: CMC, critical micelle concentration; HLB, hydrophilic–lipophilic balance; TDS, technical data sheet; BET, Brunauer–Emmett–Teller; *T*_g_, glass transition temperature.

**Table 6:** Standard deviation summary for all measurements reported in this study.

Measurement	Figure/Table	Number of Replicates (*n*)	Standard Deviation
Initial foam volume (*V*_0_)	Figure 1, Figure 5 and Figure 8; Table 3	3	±5% (mean relative SD across all concentrations tested)
Foam half-life (*T*_1_/_2_)	Figure 2, Figure 6 and Figure 9; Table 3	3	±8% (mean relative SD across all concentrations tested)
Foam quality (*Γ*)	Figure 3	3	±2% (mean relative SD)
Comprehensive foam (*F*_C_) value	Figure 4 and Figure 7; Table 3	3	±10% (mean relative SD, propagated from V_0_ and *T*_1_/_2_)
Apparent viscosity (time sweep)	Figure 10 and Figure 11	3	±5 mPa·s (typical absolute SD at plateau)
Hysteresis area	Table 5	3	±1.5 Pa·s (Foam I); ±3.2 Pa·s (Foam II); ±2.8 Pa·s (Foam III)
Structural recovery	Table 5	3	±2.1% (Foam I); ±3.5% (Foam II); ±2.9% (Foam III)
Initial permeability (*k*_i_)	Table 6	3 per fluid system	±0.15 mD (Foam I); ±0.13 mD (Foam II); ±0.16 mD (Foam III)
Final permeability (*k*_f_)	Table 6	3 per fluid system	±0.12 mD (Foam I); ±0.09 mD (Foam II); ±0.07 mD (Foam III)
Permeability damage rate (*D*_r_)	Figure 12; Table 6	3 per fluid system	±1.8% (Foam I); ±2.1% (Foam II); ±2.5% (Foam III)
Proppant settling velocity	Section 2.2.3	3	±0.02 mm/s
FC temporal stability	Section 2.2.1	3	±3.5% (Foam I)
XPS functional group abundance	Section 2.2.4	3	±2.0% (mean relative SD for C–O/C=O peaks)

## Data Availability

All data, models, or code generated or used during the study are available from the corresponding author by request.
